# Can inter-observer consistency be achieved in the laparoscopic assessment of the peritoneal carcinomatosis index score in peritoneal metastasis? A pilot study

**DOI:** 10.1515/pp-2024-0015

**Published:** 2025-04-04

**Authors:** Audrey Astruc, Valérie Seegers, Frederic Dumont, Cécile Loaec, Emilie Thibaudeau, Charlotte Bourgin, Romuald Wernert, Noémie Body, Valeria De Franco

**Affiliations:** Department of Surgical Oncology, Institut de Cancérologie de l’ouest, Angers, France; Department of Statistics, Institut de Cancérologie de l’ouest, Saint-Herblain, France; Department of Surgical Oncology, Institut de Cancérologie de l’ouest, Saint-Herblain, France

**Keywords:** peritoneal carcinomatosis, laparoscopy, PCI, reproducibility

## Abstract

**Objectives:**

The main prognostic factor for peritoneal metastasis (PM) is the complete resection of the disease during cytoreductive surgery. Accurate patient selection is therefore essential for determining eligibility for this type of surgery. The peritoneal carcinomatosis index (PCI) is a widely used tool for assessing the extent of carcinomatosis. This study aimed to evaluate the inter-observer reproducibility of PCI assessments via laparoscopy and identify factors influencing this reproducibility.

**Methods:**

Between November 2020 and November 2022, 25 laparoscopic PCI assessment videos were reviewed by six surgeons from two centers. The total PCI score, regional PCI scores, and the number of visualized PCI areas were recorded. Inter-observer concordance was analyzed.

**Results:**

The median PCI score was 12 out of 39 (range 0–39), and the median number of visualized PCI regions was 10 out of 13 (range 1–13). The intraclass correlation coefficient (ICC) for the total PCI score was 0.846 (95 % CI 0.738, 0.927). A history of abdominal surgery significantly impacted PCI assessment reproducibility (p=0.029).

**Conclusions:**

This study found a high inter-observer concordance in laparoscopic PCI assessments. Previous abdominal surgery negatively affected reproducibility, highlighting a challenge in evaluating the PCI in these patients.

## Introduction

The peritoneal carcinomatosis index (PCI) is a scoring system introduced by Sugarbaker [1] in 1995 to quantify the extent of malignancy within the peritoneal cavity. Initially validated for assessing the spread of peritoneal metastasis (PM) in gastrointestinal malignancies [2], the PCI has since been broadly applied to PM of various etiologies, particularly during laparotomy [3], [4], [5], [6]. The PCI is calculated by summing scores from 13 abdominopelvic regions (nine abdominal and four intestinal quadrants), based on the size of tumor implants and the presence confluence with individual region scores ranging from 0 to 3, yielding a total score between 0 and 39.

While the PCI is well-validated for laparotomy, preoperative assessment is essential to avoid unnecessary cytoreductive surgery. Laparoscopy has demonstrated a negative predictive value for complete cytoreduction surgery ranging from 54 to 96 %, and for optimal cytoreduction (tumor residue <1 cm) from 69 to 100 % [7] with positive predictive values between 66 and 98 % [8]. A study by Angeles et al. [9] reported good concordance between laparoscopic and laparotomic PCI evaluation.

However, visual PCI assessment can be subjective, varying with the surgeon’s experience and interpretation. Few studies have investigated the reproducibility of PCI scoring across different surgeons. The primary aim of this study was to evaluate inter-observer reproducibility of the laparoscopic PCI score and to identify factors influencing this variability.

## Methods

### Study design and population

We conducted a retrospective, multicenter study at the Paul Papin and Saint Herblain sites of the Western Oncologic Institute (ICO) in France, involving patients treated for PM between November 2020 and November 2022.

Eligible patients were aged ≥18 years, had PC of any etiology, and were candidates for diagnostic laparoscopy either prior to treatment, following systemic chemotherapy, or after pressurized intraperitoneal chemotherapy (PIPAC), with recorded video of the procedure. Patients were excluded if they declined the use of their medical data for research. Ethical approval was obtained from the Angers University Hospital Ethics Committee (no. 2023-052).

### Data collection

Intraoperative videos from laparoscopic evaluations of PM were collected, anonymized, and independently reviewed by seven experienced surgeons. Each surgeon evaluated the PCI score independently based on video review, without knowledge of the patient’s identity or the operating surgeon, though clinical details such as PC origin, age, and medical history were provided.

The primary objective was to assess inter-observer reproducibility of the total and regional PCI scores derived from laparoscopy. Secondary objectives included identifying factors that influenced reproducibility. Data collected from the patient records included: patient age, body mass index (BMI) at the time of laparoscopy, PM origin, histological type, any perioperative treatments (chemotherapy, radiotherapy), and any previous abdominal surgeries before laparoscopy. Data collected from the videos included: the PCI score (total and by region) reported by the surgeon during laparoscopy (recorded in the medical file), the PCI score calculated after anonymous video review, and a Leach adhesion score, a classification system that quantifies intra-abdominal adhesions based on their extent and density [10].

### Statistical analysis

Quantitative data were summarized using median and ranges, while categorical variables were expressed as frequencies and percentage. Missing data for regional PCI scores were not imputed; however, for the total PCI score, quadrants that could not be assessed were conservatively assumed to have “no peritoneal metastasis visualized” and assigned a score of zero, as the study focused on score comparisons rather than survival analysis.

The intraclass correlation coefficient (ICC) was used to assess inter-surgeon reproducibility for the total PCI score (continuous data), while Light’s kappa coefficient was applied to the regional PCI scores (ordinal data), as it is better suited for categorical variables and accounts for both the ordinal nature of the data and chance agreement. A higher ICC indicates greater similarity between values and thus better reproducibility. According to Conger (1980), the strength of agreement for Light’s kappa coefficient is considered very weak for values between 0 and 0.2, weak between 0.21 and 0.40, moderate between 0.41 and 0.60, strong between 0.61 and 0.80, and almost perfect between 0.81 and 1.00.

A linear regression model was used to evaluate the association between surgeon characteristics and PCI score variation (for videos viewed by all surgeons), while Spearman’s correlation and rank tests (Wilcoxon or Kruskal–Wallis) were applied to assess patient-related factors. p Values below 0.05 were considered statistically significant.

## Results

A total of 25 laparoscopic videos were reviewed, with surgeons having a median of 6 years of surgical experience [4–22], and 5 years [2–17] specifically in PM surgery (Table 1). The median patients age was 63 years, with 64 % having undergone previous abdominal surgery, and 88 % receiving neoadjuvant chemotherapy. The predominant cancer origins were ovarian (36 %) and colon (32 %), but also included gastric, breast, rectal, and small intestine origins (Table 2).

**Table 1:** Surgeons characteristics.

Characteristics	
Surgeon age (years), median	36 (35–52)
Surgical experience (years), median	6 (4–22)
Surgical experience in carcinomatosis (years), median	5 (2–17)

**Table 2:** Patients characteristics.

Baseline characteristics	
Age (years), median	63 (42–79)
BMI (kg/m^2^), median	23.2 (18.6–35.4)
PC origin^a^, n (%)	
Ovaries	9 (36)
Colon	8 (32)
Stomach	4 (16)
Breast	2 (8)
Rectum	1 (4)
Small bowel	1 (4)
Previous abdominal surgery, n (%)	16 (64)
Neoadjuvant chemotherapy, n (%)	22 (88)
Previous treatment with PIPAC^b^, n (%)	2 (16)

^a^PC origin, peritoneal carcinomatosis origin; ^b^PIPAC, pressurized intraperitoneal aerosol chemotherapy.

The median PCI score was 12/39 [0–39], with 10/13 [1–13] regions typically visualized (regions 9–10 were the less visualized) a median adhesion score of 3/10 [0–10] (Table 3). The total PCI reproducibility yielded an ICC of 0.846 (95 % CI 0.738–0.927) with weak agreement for regions 4, 5, 8 (Light’s kappa 0.35–0.38), moderate agreement for regions 6, 7, 12 (Light’s kappa 0.41–0.58) and strong agreement for the other regions (Table 4).

**Table 3:** Videos reviewing.

Characteristics	
Total PCI^a^, median	12 (0–39)
Numbers of PCI regions visualized, median	10 (1–13)
Adherence score, median	3 (0–10)

^a^PCI, peritoneal carcinomatosis index.

**Table 4:** Inter observer reproductibility analysis. According to Conger (1980), the strength of agreement for Light’s kappa is considered very weak for values between 0 and 0.2, weak between 0.21 and 0.40, moderate between 0.41 and 0.60, strong between 0.61 and 0.80, and almost perfect between 0.81 and 1.00.

PCI region	Light’s kappa coefficient	Interpretation
PCI region 0	0.61	Strong agreement
PCI region 1	0.80	Strong agreement
PCI region 2	0.76	Strong agreement
PCI region 3	0.62	Strong agreement
PCI region 4	0.37	Weak agreement
PCI region 5	0.35	Weak agreement
PCI region 6	0.44	Moderate agreement
PCI region 7	0.41	Moderate agreement
PCI region 8	0.38	Weak agreement
PCI region 9	–	
PCI region 10	–	
PCI region 11	0.71	Strong agreement
PCI region 12	0.59	Moderate agreement
All areas combined	0.64	Strong agreement

**Figure 1:**
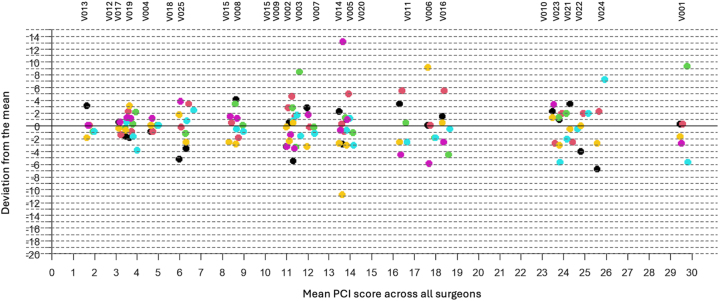
Difference between each surgeon’s PCI estimate and the mean PCI estimate across all surgeons. Each color represents a surgeon.

A history of abdominal surgery significantly influenced inter-observer agreement (p=0.029), while age, BMI, primary cancer type, history of PIPAC, and neoadjuvant chemotherapy did not. Table 5 illustrates the variability in PCI score estimates across observers, compared to the median PCI calculated from all assessments. No statistical difference was observed between the observers (p: 0.07) (Table 5, and Figure 1). Also, no correlation was observed between the surgeons’ length of experience in peritoneal metastasis surgery and the variability in PCI estimates.

**Table 5:** Difference between the surgeon’s PCI estimate and the median inter-observer PCI.

Surgeon	Mean (std) difference between the surgeon’s PCI estimate and the median inter-observer PCI
Surgeon n° 1	−0.4 (3.1)
Surgeon n° 2	+1.1 (2.4)
Surgeon n° 3	+0.3 (4.0)
Surgeon n° 4	−0.6 (2.7)
Surgeon n° 5	+0.9 (3.3)
Surgeon n° 6	−1.1 (3.4)

## Discussion

In our study, we observed a we observed a substantial inter-observer ICC of 0.846 (95 % CI 0.738–0.927). These findings are close to those reported in previous studies. For example, Gouy et al. [11], in a study involving 29 patients with ovarian PC and 16 surgeons (both junior and senior surgeons), reported a slightly higher ICC of 0.92 (95 % CI 0.86; 0.97) when evaluating PCI via laparoscopy. They found no significant difference in PCI assessments between senior and junior surgeons, nor between evaluations conducted via laparoscopy and laparotomy by the same surgeon. Similarly, Elias et al. [12] demonstrated concordance rates of 0.89 and 0.88 between experienced and less experienced surgeons in assessing total PCI and the number of affected regions, although in their study, PCI was evaluated by laparotomy.

In our clinical practice, video recordings during diagnostic or feasibility laparoscopies are left to the operator’s discretion. As a result, not all patients undergoing laparoscopy were included in this study. However, 25 high-quality videos were randomly selected between November 2020 and 2022 from two high-volume PM centers.

The inclusion of patients with varying origins and severities of PM, and a median PCI score of 12/39 (0–39), reflects a real-world patient cohort. A median of 10/13 regions were visualized, underscoring the feasibility of obtaining a comprehensive evaluation of the peritoneal cavity via laparoscopy. Furthermore, the surgeons participating in the study represented a broad range of ages and experience levels across two teams. There was no statistically significant difference between the surgeons, with the maximum mean discrepancy being 1.1/39 points. This small difference is unlikely to affect the decision to perform cytoreductive surgery in real life practice, particularly for ovarian carcinomatosis, where a PCI threshold for surgical eligibility has not been clearly established.

Cytoreductive surgery combined with intraperitoneal chemotherapy for carcinomatosis carries morbidity rates between 12 and 52 % and mortality rates of 0.9–5.8 % [13]. Therefore, accurate patient selection for this aggressive surgery is crucial. Despite advancements in preoperative imaging modalities like CT, MRI, and PET, their sensitivity for detecting lesions smaller than 5 mm remains limited [14]. The PCI score remains one of the most reliable tools for objectively quantifying disease extent [6]. Preoperative laparoscopic assessment of PCI offers several advantages, including shorter procedure time, faster postoperative recovery, reduced pain, and lower morbidity. The teams in Angers and Nantes frequently conduct feasibility laparoscopies within 15 days of planned cytoreductive surgery to streamline operating room logistics and spare patients from unnecessary surgery if resectability is not feasible.

In our study, 88 % of patients had received neoadjuvant treatment, which did not explain the variability in PCI estimates among the surgeons. According to Rawert et al. [15], the PCI score remains a reliable predictor of complete cytoreductive surgery even after neoadjuvant chemotherapy, with an AUC of 0.77 [(95 % CI 0.53–1.00), p=0.050].

However, laparoscopic PCI assessment can face challenges, particularly in the presence of adhesions that limit visualization of certain regions. In our study, prior abdominal surgery contributed to variability in PCI assessment, with a median adhesion score of 3/10. Von Breitenbuch et al. [16] reported than in 71 % of 102 diagnostic laparoscopies, the PCI could not be fully evaluated due to inadequate exploration of the peritoneal cavity, and four procedures had to be converted to laparotomy due to significant adhesions obstructing laparoscopic exploration.

This raises the question of whether certain regions are more critical to assess than others in cases where the peritoneal cavity cannot be fully explored [17]. Heitz et al. [18] noted that primary recurrence sites for ovarian carcinomatosis include the small intestine, the mesentery root, and the hepatoduodenal ligament (regions 2 and 9–12), suggesting that incomplete preoperative assessment could lead to suboptimal initial cytoreductive surgery. Rosendahl et al. [19] similarly found that failure to achieve CC0 resection in 80 % of cases was due to involvement of regions 2 and 9–12. They further demonstrated that these regions were more predictive of complete surgery than the total PCI score (AUC of 79 vs. 75 % for total PCI) and were associated with poorer survival outcomes (HR: 1.08; 95 % CI: 1.06–1.11). Access to these regions can be challenging during laparoscopy, potentially leading to disease underestimation.

Improving reproducibility in PCI assessment would enable a more objective evaluation of PM, fostering a standardized approach that minimizes surgeon subjectivity. As Artificial Intelligence becomes increasingly integrated into surgical practice, establishing standardized criteria for laparoscopic PCI evaluation – such as the number or location of regions to be explored – could significantly enhance accuracy and consistency.

## Conclusions

This study highlights the substantial reproducibility of PCI assessment via laparoscopy, emphasizing the impact of prior abdominal surgery on visualization and scoring. The ICC of 0.846 suggests a high level of agreement among observers, reinforcing the reliability of laparoscopic PCI evaluation. Further research is required to refine laparoscopic techniques, improve inter-observer consistency, and correlate visual macroscopic evaluations with histopathological findings. While no universally accepted PCI threshold has been established, improving the accuracy of PCI assessment may help identify specific thresholds that could provide valuable guidance for selecting candidates for cytoreductive surgery and intraperitoneal chemotherapy.

## Supplementary Material

Supplementary Material
