# Supplementary figures and images for: Can inter-observer consistency be achieved in the laparoscopic assessment of the peritoneal carcinomatosis index score in peritoneal metastasis? A pilot study
Source: Pleura Peritoneum. 2025 Apr 4;10(1):19–23. doi: 10.1515/pp-2024-0015 (PMC12016015; doi:10.1515/pp-2024-0015)

**Supplementary Material**

Supplementary Figure: The adherence score: the Leach Score


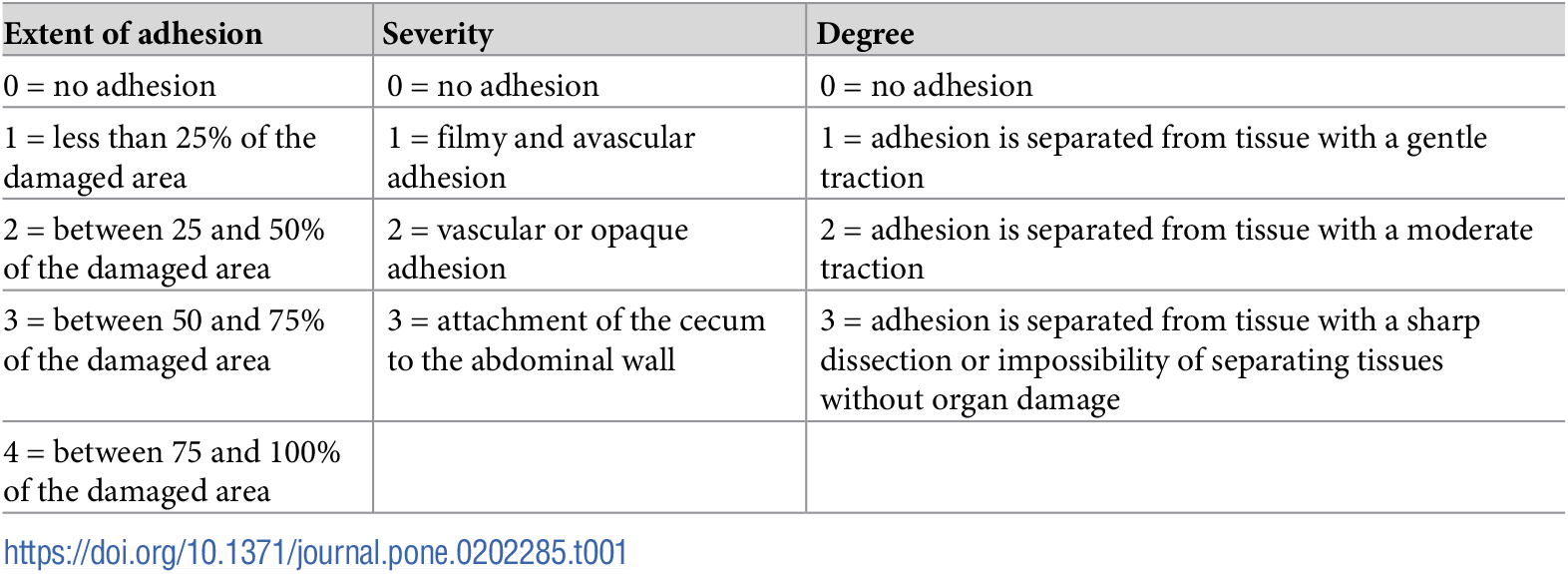

Supplement: Supplementary file 1 — Supplementary Material [file j_pp-2024-0015_suppl_001.docx]
